# His_1_-tagged DM or DDM detergent micelles are reversibly conjugated by nickel ions

**DOI:** 10.1038/s41598-023-44236-x

**Published:** 2023-10-10

**Authors:** Mitra Lal, Ellen Wachtel, Soumyaranjan Pati, Irishi. N. N. Namboothiri, Guy Patchornik

**Affiliations:** 1 Department of Chemical Sciences, Ariel University, 70400 Ariel, Israel; 2 Faculty of Chemistry, Weizmann Institute, 761001 Rehovot, Israel; 3 Department of Chemistry, Indian Institute of Technology Bombay, Powai, Mumbai, 400076 India

**Keywords:** Structural biology, X-ray crystallography

## Abstract

Specific conjugation of decyl β-D-maltoside (DM) or dodecyl β-D-maltoside (DDM) detergent micelles is accomplished between pH 7.0–8.5 in the presence of an amphiphilic analog of the amino acid histidine, bound to a 10-carbon hydrocarbon chain (His_1_-C10) and Ni^2+^ ions. Following addition of 10–15 wt% PEG-6000 as precipitant, phase separation in the form of oil-rich globules (30–600 µm) is observed by light microscopy. Other divalent cations: Zn^2+^, Fe^2+^, Cu^2+^ lead to dark precipitates rather than colorless globules; while Mg^2+^, Ca^2+^ do not promote any phase separation at all. Even in the absence of precipitant, dynamic light scattering (DLS) measurements demonstrate that DM micelles (hydrodynamic size ~ 6 nm) or DDM micelles (8 nm) self-associate into larger particles (9 nm and 411 nm for DM; 10 nm and 982 nm for DDM) in the presence of His_1_-C10 and nickel ions. Micellar conjugation is partially reversible in the presence of water soluble 50 mM EDTA, histidine or imidazole chelators. Cryo-transmission electron microscopy (cryo-TEM) imaging revealed the formation of non-uniformly dense detergent aggregates for both DM and DDM micelles in the presence of precipitant. The possible utility of such His_1_-tagged DM or DDM micelles for promoting crystallization of integral membrane proteins is discussed.

## Introduction

The growth of high quality, three-dimensional crystals remains a bottleneck in the classical determination of protein structure. To circumvent this problem, at least for proteins with MW > 100 kDa, single-particle electron cryo-microscopy (cryo-EM) has become, during the last decade, an alternative method of choice for protein structure determination^1,2^. To date, more than 200,000 high resolution structures of water-soluble proteins have been deposited in the Protein Data Bank (PDB)^3^. However, structural determination of integral membrane proteins (MPs), small proteins generally characterized by MW < 100 kDa, is even more challenging^4,5^. Synthetic detergents, above their critical micelle concentration (cmc), must be used to efficiently extract MPs from the biological membrane, and to maintain solubility in aqueous media throughout the crystallization process^5,6^. Although extraction of membrane proteins from phospholipid membranes is best achieved with ionic detergents^7,8^, preservation of the native, functional state of membrane proteins following extraction is optimal with non-ionic detergents^9^. In spite of the physiological significance of MPs, only ~ 3000 structures have so far been determined^10^.

In crystallization trials, an integral MP is embedded in, and surrounded by, a detergent micelle;^4,11^ together they constitute a non-covalently bound, protein-detergent complex (PDC). While non-ionic detergent micelles have primarily served as a passive shield against membrane protein denaturation and/or aggregation in aqueous solution, we have focused on exploiting the physical chemistry of such micelles in order to direct PDC's to assemble via conjugation under ambient conditions. Detergents which have been particularly successful in this regard include DM and DDM, both with sugar-rich hydrophilic headgroups^11^. It has long been recognized that establishing favorable crystallization conditions for PDCs requires weakly attractive intermicellar interactions under conditions which promote native MP conformation^12–16^. Only in that way would the essential conformational flexibility be provided for stable crystal nuclei to form. Such an objective may sometimes be achieved by the inclusion of precipitants, including polymers (*e.g.,* polyethylene glycol, PEG); inorganic salts (*e.g.,* ammonium sulfate) or low molecular weight organic molecules^17^, that lower the water solubility of all components in the system.

Our group has sought a way to improve the fine tuning of intermicellar interactions by focusing on amphiphilic metal ion chelators. Among the parameters which must be optimized are pH, temperature, metal ion species and ion concentration, while at the same time, solution conditions must remain non-denaturing for the MPs. We have demonstrated that amphiphilic [divalent cation:chelator] complexes, generated at the micelle\water interface, are able to conjugate micelles under non-denaturing conditions and to generate micron size micellar aggregates^18,19^, even in the absence of precipitants. Strong metal chelators such as 1,10-phenanthroline or bipyridine analogs have been tested, both in the absence and presence of MPs. However, the protein crystals generated were either too small or too thin to give useful X-ray diffraction, perhaps due to the overriding strength of the intermicellar interactions^20^.

**Figure 1:**
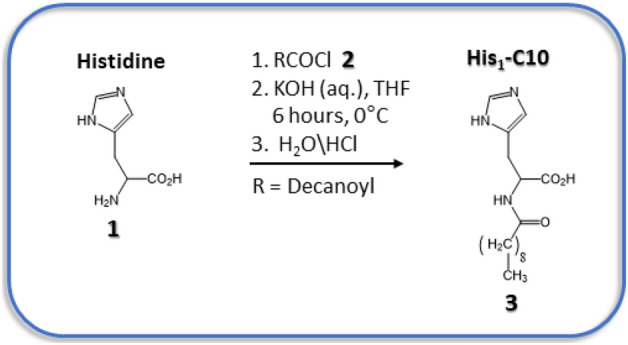
Synthesis of the amphiphilic His_1_-tag derivative labelled His_1_-C10 (product **3**).

**Figure 2:**
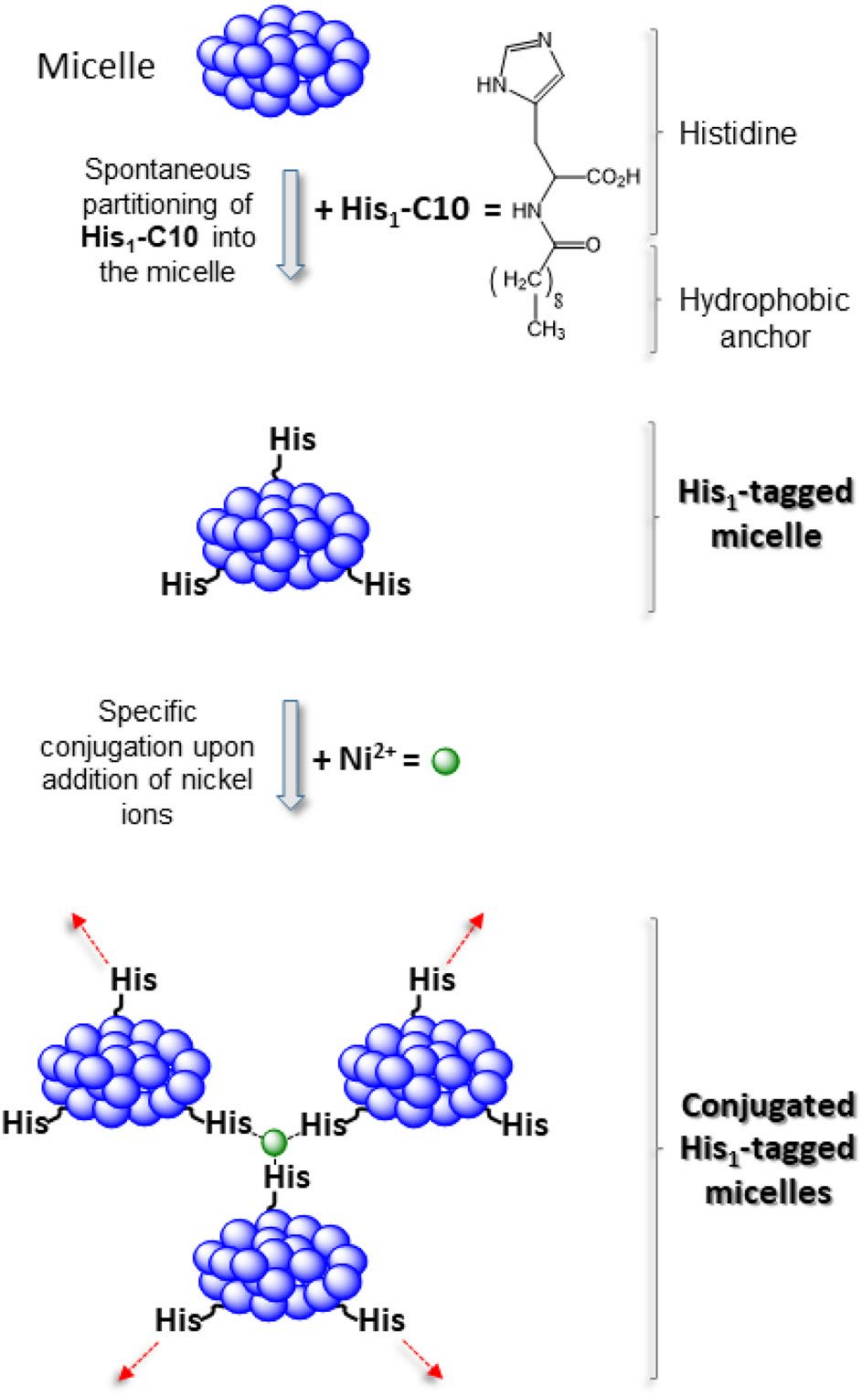
Cartoon demonstrating how DM or DDM micelles may be conjugated via nickel ion chelation using the amphiphilic histidine derivative His_1_-C10.

In the present work, we describe specific conjugation of DM or DDM micelles, accomplished at 19 °C, pH 7.0–8.5 via a purpose synthesized, amphiphilic analog of the amino acid histidine, bound to a 10-carbon hydrocarbon chain (termed His_1_-C10; Fig. 1), which is able to chelate Ni^2+^ ions (Fig. 2). We suggest that the partial reversibility of micelle conjugation observed in the presence of competing, water soluble metal chelators, may potentially provide sufficient flexibility to allow fine tuning and to promote stable MP crystal nucleation.

## Experimental

### Materials

Decyl β-D-maltoside (DM) (Sigma 850521P, 99% pure); *n*-dodecyl-β-D-maltoside (DDM) (Sigma, D4641, 98% pure); *L*-histidine (Sigma H8000, 99% pure); imidazole (Sigma I202, 99% pure); NiCl_2_ (Sigma, 339,350 98% pure); ZnCl_2_ (Sigma, 208,086, 98% pure); CuCl_2_ (Sigma, 751,944, 99% pure); FeCl_2_ (Sigma, 372,870, 98% pure); EDTA (Sigma, 03,609, 99% pure); PEG-6000 (Sigma, 89,510), Tris (Sigma, 93,352, 99% pure); tetrahydrofuran (THF) (Sigma, 186,562, 99.9% pure); decanoyl chloride (Sigma, 140,295, 98% pure); KOH (Sigma, 757,551, 99% pure).

## Methods

### Synthesis of ***N***-decanoyl-L-histidine (His_1_-C10)^21^

To a stirred solution of *L*-histidine **1** (930 mg, 6 mmol) and KOH (350 g, 6 mmol) in 100 ml of water and 20 ml of THF, acid chloride **2** (1.3 mL, 6 mmol) in 20 ml of THF was added dropwise over 1.5 h at 0 °C. This mixture was vigorously stirred during addition continuing for 6 h at the same temperature. THF was then removed and the aqueous layer was washed thoroughly with *n*-hexane (3 × 50 mL) to remove the unreacted decanoyl chloride **2**. Next, the aqueous layer was acidified with formic acid and the pH was adjusted to 5–6. The residue thus obtained was filtered and washed thoroughly with water and dried under vacuum. The resulting residue was recrystallized from hot water to produce the desired *N*-decanoyl-histidine **3** as an off-white amorphous solid (650 mg, 35% yield) (see Fig. 1). Details of the NMR spectrum of His_1_-C10 are presented in Supplementary Section S1.

### Conjugation of DM and DDM micelles via amphiphilic His_1_-C10 and Ni^2+^

Solution A was prepared by the sequential addition of: 6 µL of 100 mM DM (in double distilled water (DDW)); 1 µL of 75 mM His_1_-C10 (in methanol); 8 µL of 300 mM Tris (pH 8); 6 µL of 25 mM NiCl_2_ in DDW; 9 µL of DDW; and further incubated for 15 min at room temperature. 1.5 µL of Solution A was mixed with solution B containing 1.5 µL of 30 wt% PEG-6000 in DDW on siliconized cover slips. The mixture was then incubated for 1 h at 19 °C over a reservoir (0.5 mL) containing 30 wt% PEG-6000 in VDX crystallization plates (Hampton Research). An identical protocol was used for conjugating DDM micelles where the concentration of DDM in stock solution A was 100 mM and Solution B contained 20 wt% PEG-6000 in DDW. The final molar ratio of His_1_-C10 to Ni^2+^ was greater than 1.

### Light microscopy

Images of hanging drops were obtained using an Olympus CX-40 light microscope equipped with an Olympus U-TV1X-2 digital camera.

### Dynamic light scattering

Samples for dynamic light scattering (DLS) measurements (0.4 mL final volume) were prepared by dispersing 20 μL of 0.2 M DM or DDM (in DDW) in an additional 380 μL DDW. This was followed by 10 min of centrifugation at a relative centrifugal force of 21,000 prior to analysis at 19 °C. Micellar conjugation was performed as described above. PEG-6000 was not present. Intensity-weighted particle size distributions were determined using the auto correlation spectroscopy protocol of a NANOPHOX instrument (Sympatec GmbH, Germany).

### Partial reversibility of micelle conjugation by water-soluble chelators

Micellar reversibility was studied both by light microscopy and by dynamic light scattering (DLS). In the case of the former, conjugated His_1_-tagged DM or DDM micelles were prepared in the presence of PEG-6000, as described above, followed by the addition of 50 mM histidine. In the case of the latter, conjugated His_1_-tagged DM or DDM micelles were prepared without PEG-6000, followed by the addition of 50 mM imidazole or EDTA.

### Cryo-TEM imaging

Conjugated His_1_-tagged DM and DDM micelles were prepared with added PEG-6000 as described above, and subjected to cryo-TEM imaging. Cryo-TEM sample preparation was as follows. Four microliters of sample were deposited on Quantifoil copper grids that had been glow-discharged to produce a charged surface. The grid was then blotted and plunged into liquid ethane using a Leica EM-GP™ rapid freezing device. Grids were imaged with an Arctica microscope (Thermo Fisher Scientific), equipped with a zero-loss energy filter (Gatan) and a Gatan K2 direct electron detector.

## Results

### His_1_-C10 / Ni^2+^ complexes conjugate DM or DDM micelles

**Figure 3:**
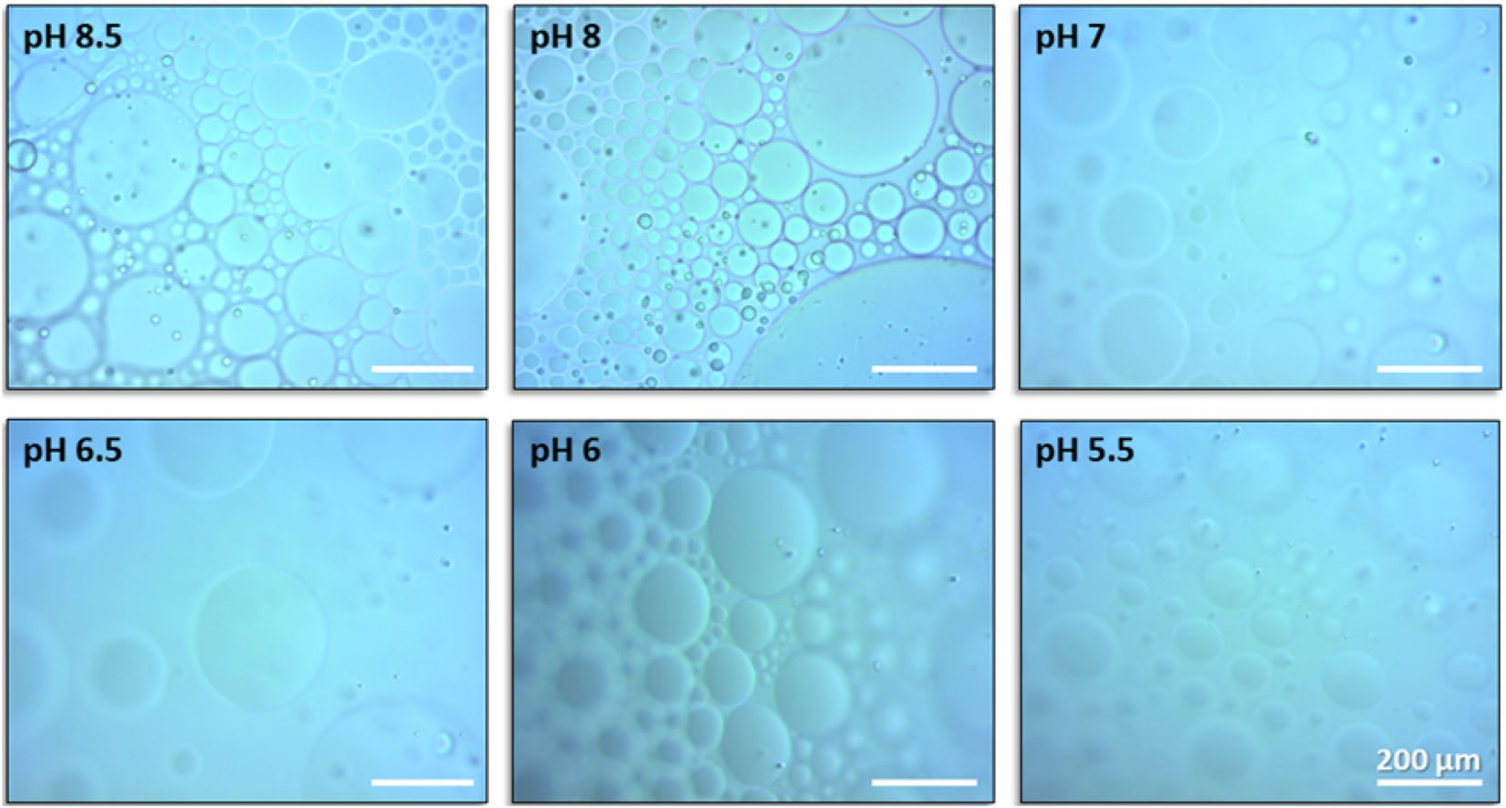
Phase separation of conjugated His_1_-tagged DM micelles with chelated Ni^2+^ ions as a function of pH. Light microscope images of 10 mM DM incubated with 1 mM His_1_-C10, 2.5 mM Ni^2+^ 15wt% PEG-6000 in 40 mM Tris buffer at different pH values following 1 h incubation at 19 °C. All scale bars indicate 200 mm.

In previous reports^18–20,22^, we have shown that strongly interacting amphiphilic [metal chelator:divalent cation] complexes, such as [1,10-phenanthroline or bipyridine analogs:Fe^2+^] are able to conjugate DM or DDM micelles, both in the absence and presence of integral membrane proteins. The dissociation constant for Fe^2+^ bound to 1,10-phenanthroline is K_d_ ~ 10^–21^ M^23,24^. As such, the conjugated DM or DDM micellar aggregates were very condensed (Supplementary-Fig. S1). No precipitant was required for micron-scale, dense aggregates to form; however, the membrane protein crystals generated were either too small or too thin to provide useful X-ray diffraction data^20^. In the current work, we sought to move closer to our goal of fine-tuning the intermicellar interactions. Only in that way would the necessary conformational flexibility be provided for stable crystal nuclei to form. Indeed, incubation of DM micelles at 19 °C for 60 min with, at least, equimolar amounts of the amphiphilic histidine derivative His_1_-C10 and Ni^2+^ leads to the appearance in the light microscope of colorless, oil-rich, globules (5–400 µm in diameter) only in the presence of 15 wt% PEG-6000 (Fig. 3).

Although K_d_ has not yet been determined for this chelator/cation pair, our observation does provide evidence for the relatively weak binding affinity between His_1_-C10 and nickel ions. Globule formation is observed when the stochiometric ratios between detergent and His_1_-C10 (Fig. 3) is 10:1 and between His_1_-C10 and Ni^2+^, 1: < 10 (Supplementary, Fig. S2). Together with the broad range of literature aggregation numbers (N_A_)^22–27^ of DM and DDM micelles (86–103 and ~ 135, respectively; also, Table 1), and the well-established cmc values of DDM and DM (0.17 and 1.8 mM, respectively)^24,25^, it is possible to estimate that ~ 8 His_1_-C10 chelators participate, on average, in each DM micelle and ~ 14 chelators in each DDM micelle.

**Table 1:** Physical chemical characteristics of DM and DDM detergent micelles under ambient conditions.

	Detergent MW (Dalton)	N_A_	cmc (mM)	Cloud point	Triaxial micelle model semi-axes (nm)^26,27^	[His_1_-C10: detergent] input molar ratio	[His_1_-C10] per micelle
DM	482.5	70–90	1.8	Unknown, but well above ambient	2.5; 3.25; 2.0	1: 10	~ 8
DDM	510.6	~ 135	0.17	Unknown, but well above ambient	3.72; 3.0; 2.2	1: 10	~ 14

N_A_ is the aggregation number (not known with great precision); cmc is the critical micelle concentration; cloud point is the temperature at which the micellar dispersion is no longer transparent and isotropic.

For reasons we do not as yet understand, only nickel ions lead to the appearance of oil-rich globules whereas Zn^2+^, Fe^2+^ and Cu^2+^ generate dark brown-colored precipitates; Mg^2+^ or Ca^2+^ do not promote any phase separation at all (Supplementary Fig. S3). While large-scale phase separation of His_1_-tagged DM micelles required the presence of at least 15wt% PEG-6000, His_1_-tagged DDM required no more than 10 wt%. We tentatively suggest that the difference in the minimum required precipitant concentration may derive from the order of magnitude difference in detergent cmc or differences in micelle dimensions (Table 1).

### pH and the binding site for Ni^2+^

In order to probe the apparent inability of the His_1_-C10 chelators to bind divalent cations other than Ni^2+^, we sought to determine whether the free carboxylic acid of His_1_-C10, it's imidazole side chain or both, participate in Ni^2+^ chelation. Control experiments were performed as described in the Methods section (*i.e.,* 10 mM DM or DDM, 2.5 mM Ni^2+^, pH 7–8.5, 15wt% PEG-6000), except that His_1_-C10 was replaced by 10 mM decanoic acid. Consequently, the imidazole moiety was lacking. Micron size, oil-rich globules were not observed in the light microscope (data not shown), implying that the imidazole ring of His_1_-C10 is necessary for micellar conjugation with or without the assistance of the free carboxylic acid. We found that conjugation of His_1_-tagged DM micelles is accomplished between pH 5.5 to 8.5 (Fig. 3). With more acidic pH values (pH < 5), oil rich globules are not observed in the light microscope (data not shown). Their absence is presumably due to protonation of the imidazole ring thereby preventing the electron pair of the nitrogen atom from participating in metal chelation.

### Dynamic light scattering and conjugation reversibility

**Figure 4:**
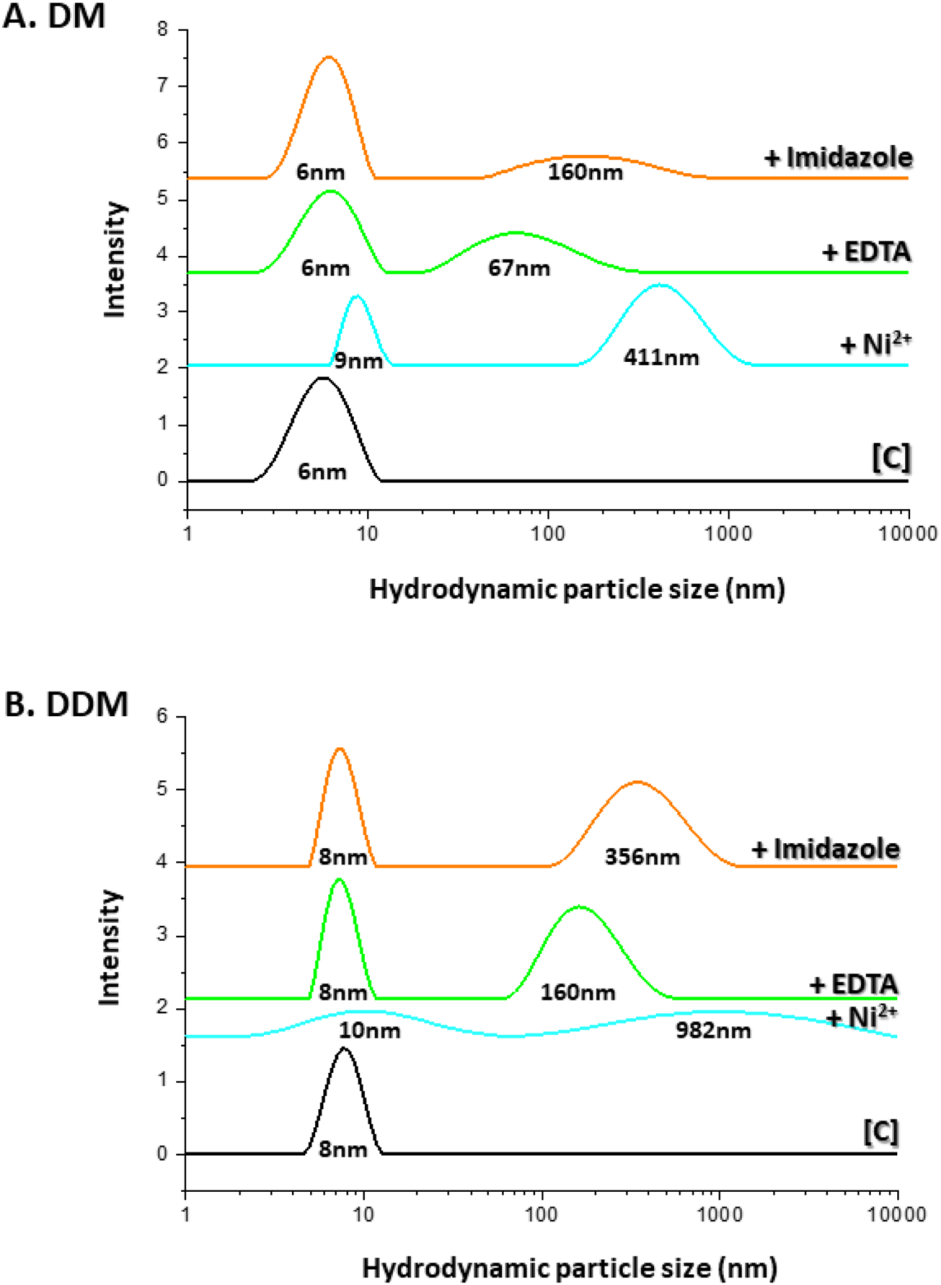
Partial reversibility of the conjugation process as monitored by DLS. (**A**–**B**) Black line: individual DM or DDM micelles at detergent concentration 10 mM, as control [C]; Cyan line: 10 mM DM or DDM, 30 min after addition of equimolar amounts (1 mM) of His_1_-C10 and Ni^2+^ at pH 8, 19 °C. Green and orange lines, respectively: effect of addition of either 50 mM EDTA or 50 mM imidazole, 30 min after DM or DDM conjugation. PEG-6000 is not present; however, aggregates approx. 0.5–1 micron in size form upon micelle conjugation even in the absence of PEG. Curves are translated vertically for ease of viewing.

To promote stable nucleation centers for protein-detergent complexes (PDCs), (but not overly stable), micellar conjugation must be controllable/reversible. Hydrodynamic particle size distributions found by dynamic light scattering (DLS), which examines the submicron, rather than the 100 μm to mm range, were consistent with light microscopy images. The hydrodynamic size of independent (control) DM micelles (10 mM detergent) was found to be 6 nm whereas that of independent (as control) DDM micelles (10 mM) was 8 nm^25^. We note that in the absence of Ni^2+^ ions, the partitioning of His_1_-C10 into the DM or DDM micelles leaves the hydrodynamic size of the independent micelles essentially unchanged (Supplementary, Fig. S4). However, the addition of both 1 mM His_1_-C10 and 2.5 mM Ni^2+^ resulted in a dramatic increase in particle size for both DM and DDM micelles (Fig. 4A–B). Two particle populations with sizes 9 nm and 411 nm were generated in the case of DM; 10 nm and 982 nm, in the case of DDM. The PEG precipitant was not present.

**Figure 5:**
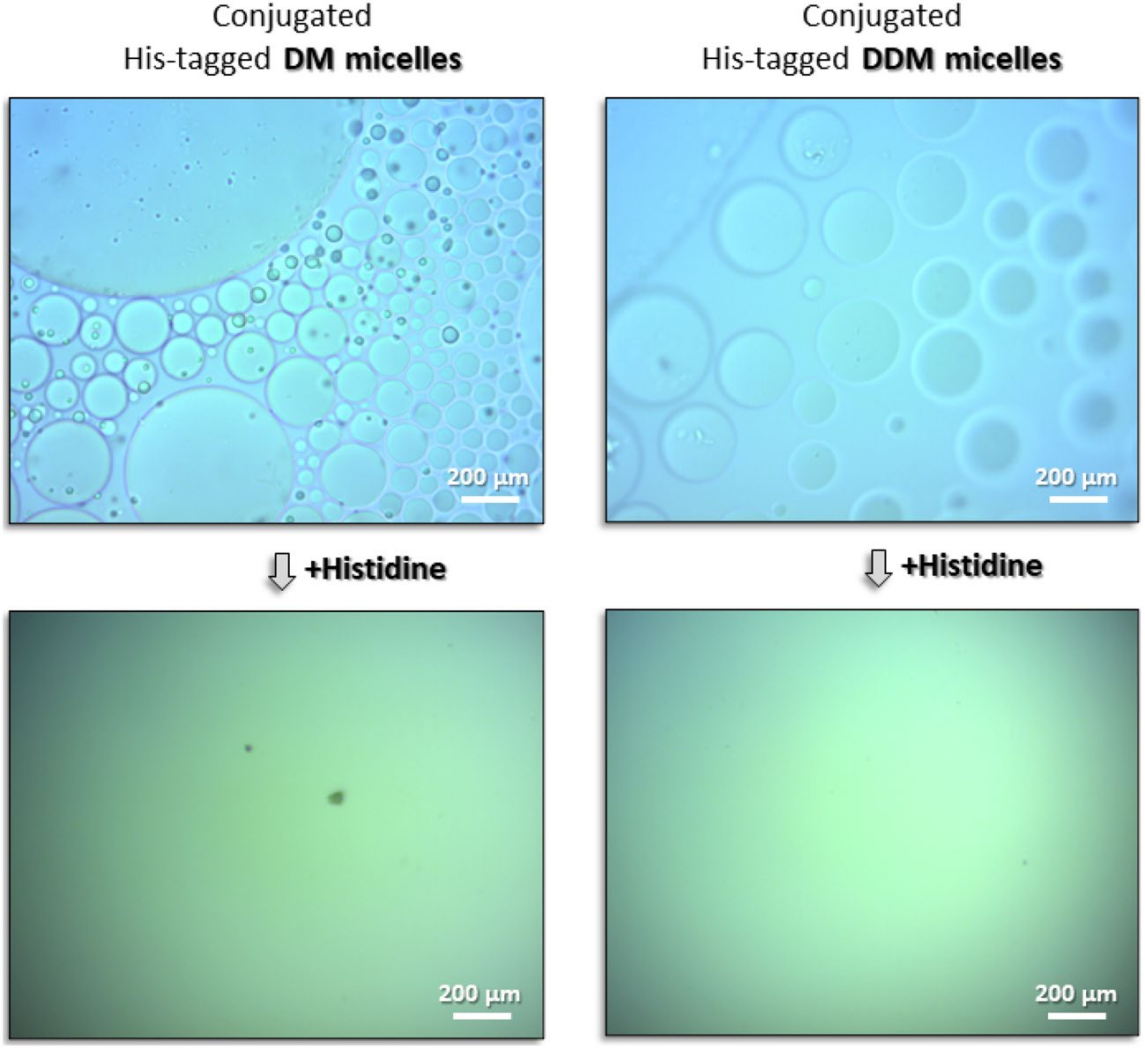
Upper panels: Light microscope images of phase separation of oil rich globules containing conjugated DM or DDM micelles (detergent concentration 10 mM), 15 wt% PEG-6000, 30 min after addition of 1 mM His_1_-C10 and 1 mM Ni^2+^ at pH 8, 19 °C; Lower panels: effect of the addition of 50 mM histidine monomers at pH 7, 19 °C after additional 30 min incubation.

These findings provide direct insight at the micellar level, as to the ability of the [His_1_-C10:Ni^2+^] complex to generate aggregates that are orders of magnitude larger than individual DM or DDM micelles. And yet, the large-scale aggregation is partially reversible. This was demonstrated by including either strong (ethylenediaminetetraacetic acid, EDTA), or weak (imidazole), water soluble chelators in the DLS sample. We found that addition of 50 mM EDTA to conjugated His_1_-tagged DM micelles, led to the reappearance of the 6 nm micellar peak; an additional peak appeared at 67 nm (Fig. 4A). It is unclear to us why such a strong chelator as EDTA was not able to completely reverse the conjugation; more study is obviously required. Repetition of the measurement with 50 mM imidazole was similarly able to recover the independent peak of DM micelles and an additional population of particles at 160 nm (Fig. 4A). Analogous findings were obtained with conjugated His_1_-tagged DDM micelles (Fig. 4B). On the macroscale, the disappearance of phase separated, oil rich globules was observed in the light microscope within minutes following addition of 50 mM histidine to both conjugated His_1_-tagged DM or DDM micelles (Fig. 5). We may conclude on this basis that process reversibility is efficient, but certainly not quantitative, as evidenced by DLS.

### Cryo-TEM imaging

**Figure 6:**
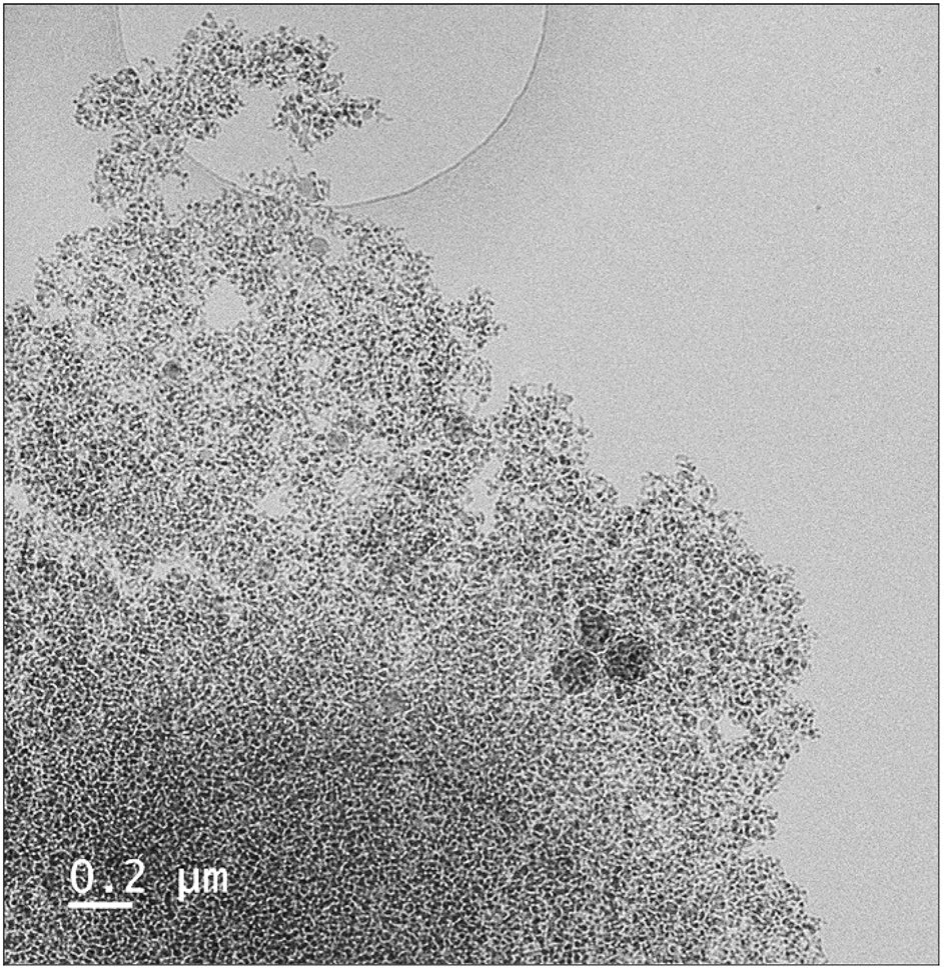
Cryo-TEM image of DDM micelles (10 mM detergent) conjugated with 1 mM His_1_-C10 and 2.5 mM Ni^2+^ ions in 40 mM Tris buffer (pH 8), 15 wt% PEG-6000 following overnight incubation at 19 °C. The black-colored dots indicate the location of heavy metals. Average center to center distance is estimated to be approx. 16 nm.

Cryo-transmission electron microscopy (cryo-TEM) allows imaging of conjugated micelles, hundreds of nm in size, rapidly frozen in thin, amorphous ice. Conjugated DDM micellar aggregates (Fig. 6) appear non-uniformly dense; while the presence of heavy metals, presumably Ni, (black spots) can be used as a ruler to estimate inter-micelle distances. These distances (approx. 16 nm) are in reasonable agreement with modeling of the DDM oblate ellipsoidal micelle structure (Table 1)^26,27^.

## Discussion

For this study, we synthesized an amphiphilic histidine derivative containing a 10-carbon tail (His_1_-C10) and assessed its ability to conjugate two of the most successful non-ionic detergents used for crystallization of integral MPs, decyl- β-D-maltoside (DM) and dodecyl-β-D-maltoside (DDM)^11^. We suggest that the driving force for micellar conjugation is derived from two chemical properties: (i) each His_1_-C10 amphiphile contains two functional groups, both of which may participate in metal chelation. These groups are the free carboxylic acid—which is likely deprotonated and hence negatively charged at pH > 5.5 and an imidazole side chain, with a pK_a_ of ~ 6^28^ that would be essentially neutral, even at pH 6, and consequently also able to participate in metal binding; (ii) the partitioning of 8–14 His_1_-C10 amphiphiles into DM or DDM micelles (Table 1). Therefore, each micelle may provide several chelating groups to support formation of a [(His_1_-C10)_x_:Ni^2+^] complex, where x > 1. Although it has been demonstrated that significant binding affinity towards nickel ions requires a sequence of 6 histidines^29^, such sequences do not contain negatively charged carboxylates that may also participate in and strengthen complex formation. Consequently, a sequence of histidines can only rely on the number and orientation of imidazole rings, which constitute a fundamentally different chemical environment from the one presented here**.**

**Figure 7:**
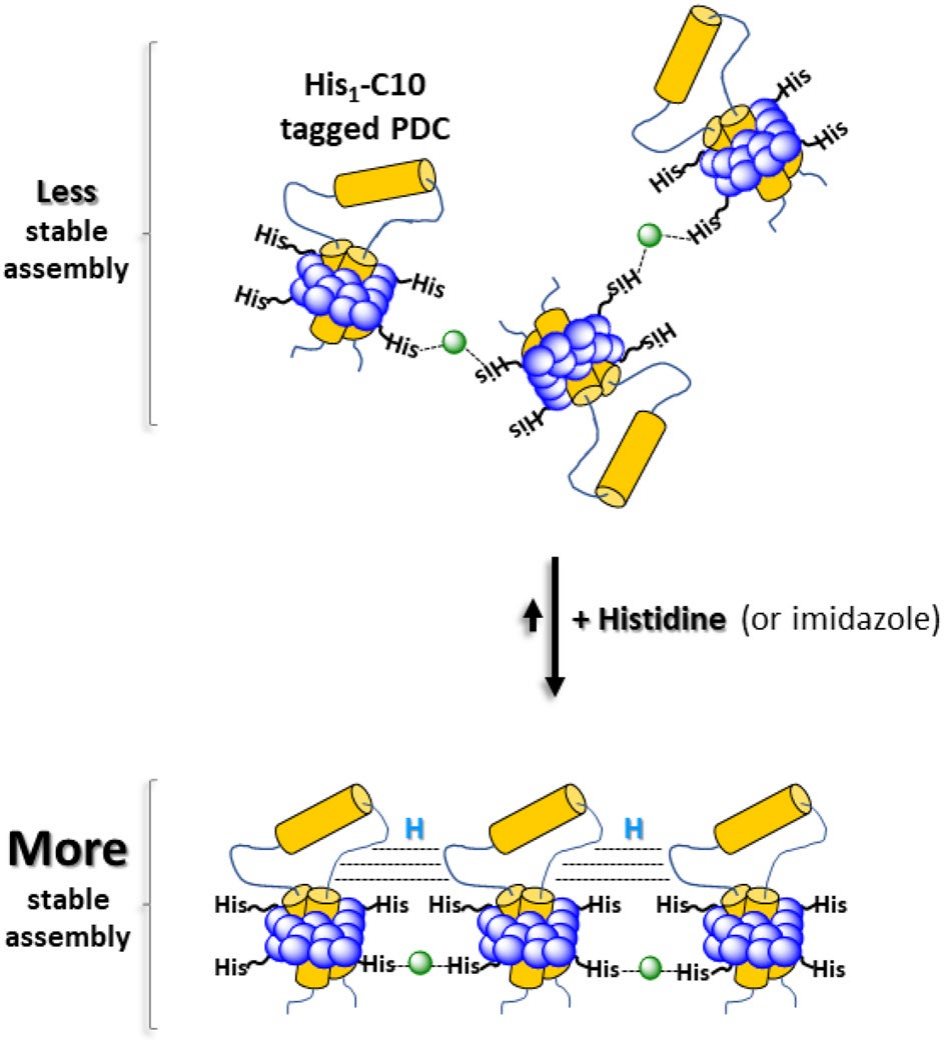
Cartoon showing how His_1_-C10 tagged DM or DDM micelles, encapsulating a membrane protein (PDC- protein detergent complex), may be conjugated via Ni^2+^ ions (small green spheres). In the presence of added, low concentrations of histidine monomers or imidazole, association\dissociation events are energetically feasible, and take place until a stable, ordered nucleation assembly, is achieved. The cartoon is not to scale.

In Fig. 7 we present a cartoon describing how amphiphilic His_1_-C10 tagged DM or DDM micelles, each containing an encapsulated membrane protein (PDC-protein detergent complex), may be conjugated via chelated Ni^2+^ ions (small green spheres). Potential interference with the proposed micellar-conjugation mechanism might derive from the presence of surface-exposed, histidine amino acid residues of the target membrane protein. These may compete with amphiphilic His_1_-C10 for binding to nickel cations, thereby weakening micellar conjugation; alternatively, the membrane protein might participate in metal cation binding in parallel with the amphiphilic chelator, thereby stabilizing intermicellar interactions. Assuming that temporal and spatial disorder within the conjugated micelle aggregate are initially present in solution, the addition of controlled concentrations of histidine monomers, or its imidazole moiety, should permit energetically feasible, association\dissociation events until, with time, a stable, ordered crystal nucleation assembly, is achieved.

### Supplementary Information

Supplementary Information.

## Data Availability

The datasets used and/or analysed during the current study are available from the corresponding author upon request.
