# Supplementary material for: His1-tagged DM or DDM detergent micelles are reversibly conjugated by nickel ions
Source: Sci Rep. 2023 Oct 10;13:17138. doi: 10.1038/s41598-023-44236-x (PMC10564902; doi:10.1038/s41598-023-44236-x)
Supplement: Supplementary file 1 — Supplementary Information. [file 41598_2023_44236_MOESM1_ESM.docx]

**Supplementary Information**

**for**

**His_1_-tagged DM or DDM detergent micelles are reversibly conjugated by nickel ions**

Mitra Lal ^1^, Ellen Wachtel ^2^, Soumyaranjan Pati ^3^,

Irishi. N. N. Namboothiri ^3^ and Guy Patchornik ^1*^

^1^ Department of Chemical Sciences, Ariel University, 70400, Ariel, Israel.

^2^ Faculty of Chemistry, Weizmann Institute, Rehovot 761001, Israel.

^3^ Department of Chemistry, Indian Institute of Technology Bombay, Powai, Mumbai

400076, India.

*Corresponding author:

Email: [guyp@ariel.ac.il](mailto:guyp@ariel.ac.il)

**Section S1.** **NMR spectral analysis** of (*S*)-2-decanamido-3-(1*H-*imidazol-4-yl)propanoic acid (compound **3** in **Figure 1**). Off white Solid; Yield 650 mg, 35%; mp 131-133 ºC; IR (KBr, cm^-1^) 3153 (s), 2922 (vs), 2851 (vs), 1725 (s), 1714 (s), 1635 (vs), 1601 (vs), 1466 (m), 1399 (vs), 1252 (m), 1181 (m), 851 (m), 628 (m); ^1^H NMR (Acetone-d_6_, 400 MHz) δ 0.92 (t, *J* = 8.3 Hz, 3H), 1.32-1.36 (unresolved m, 12H), 1.64 (quint, *J* = 9.1 Hz, 2H), 2.27 (t, *J* = 9.1 Hz, 2H), 3.02-3.04 (unresolved m, overlaps with H_2_O, 1H), 3.35-3.36 (unresolved m, 1H), 4.61, 4.65 (ABqd, *J* = 14.8 Hz, the upper half and lower half are further split into d with *J* = 8.5 and 6.0, respectively, 2H), 7.06 (s, 1H), 7.62 (d, *J* = 8.5 Hz, 1H), 7.82 (s, 1H); ^13^C NMR (D_2_O, 100) δ 13.7, 22.5, 25.5, 27.6, 29.1, 29.2, 29.3, 29.4, 31.8, 35.9, 54.2, 116.7, 130.1, 133.1, 175.1, 176.5; HRMS (ES+) calculated for C_16_H_28_N_3_O_3_ (MH^+^) 310.2125, found 310.2124.


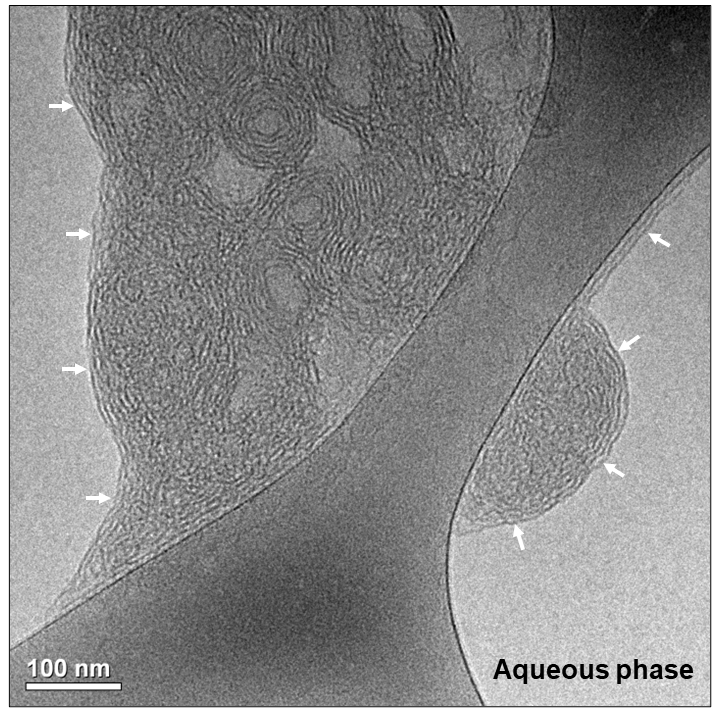


**Figure S1:** Cryo-TEM image of DDM micelles (1.25 mM detergent) conjugated with 1 mM [(bathophenanthroline)_3_:Fe^2+^] complex. [1] Arrows indicate well-defined amorphous ice /detergent micellar aggregate interface. Scale bar – 100 nm.

**
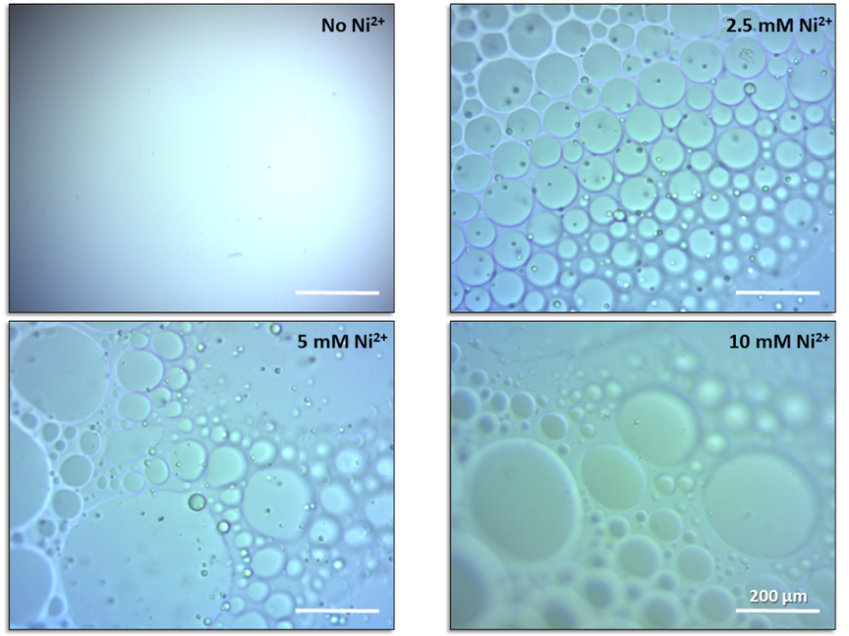
**

**Figure S2**: Effect of Ni^2+^ concentration on conjugation of His_1_-tagged DM micelles. Light microscope images of phase separation of oil rich globules containing 10 mM DM detergent, incubated for 1 h at 19°C with 1 mM His_1_-C10, 0-10 mM Ni^2+^, 15 wt% PEG-6000 in 40 mM Tris buffer, pH 8. The scale bar in all panels indicates 200 μm.


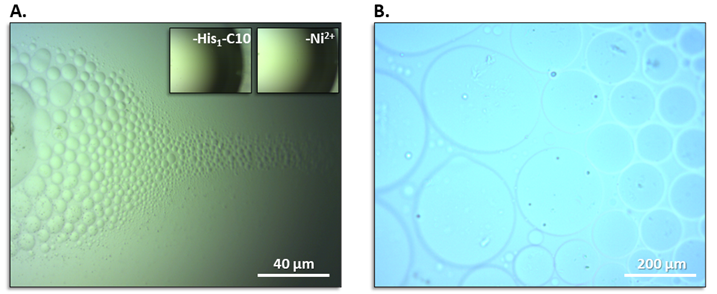


**
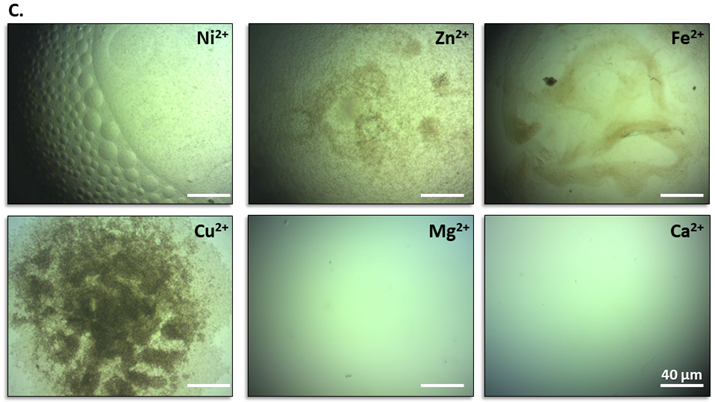
**

**Figure S3:** **A.** Light microscope images of DDM (10 mM detergent) incubated with 1 mM His_1_-C10, 10 wt% PEG-6000 and 2.5 mM Ni^2+^ in 40 mM Tris buffer at pH 8 following 1 hour incubation at 19°C. Inset shows control samples devoid of either the amphiphilic chelator (His_1_-C10) or Ni^2+^ cations; **B.** Magnification of **A**; **C**. As in **A**, but with 2.5 mM divalent cations other than Ni^2+^.


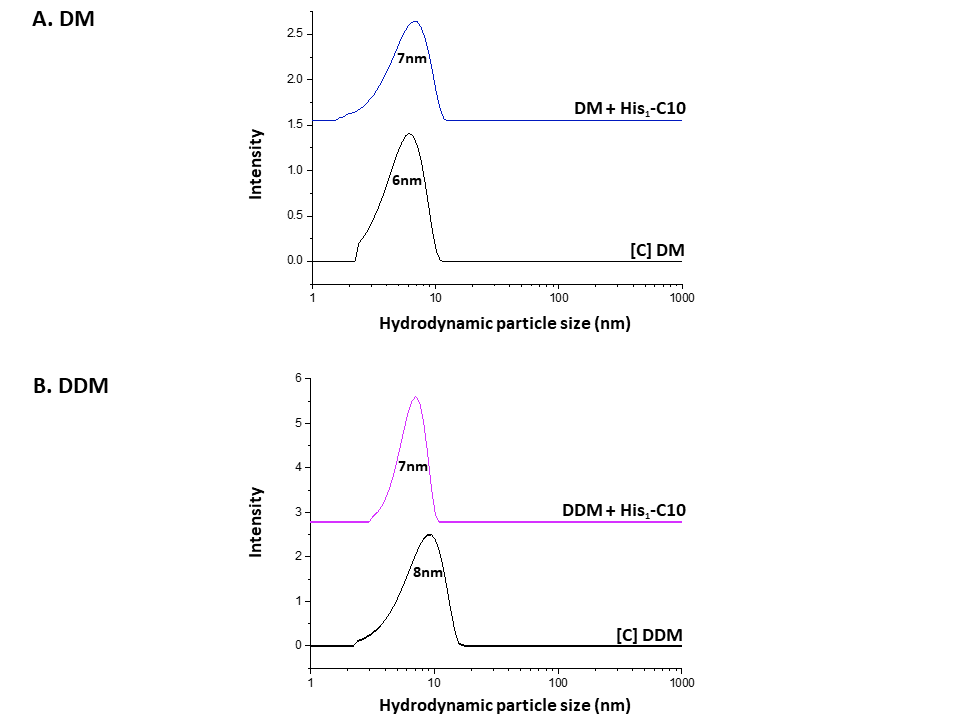


**Figure S4: Effect of His_1_-C10 on the hydrodynamic size distribution of DM or DDM micelles.** Black curves in **A** and **B** represent individual DM or DDM micelles at detergent concentration 10 mM, as control [C]; Blue and magenta curves in **A** and **B**, respectively, represent the system 30 minutes after addition of 1mM His_1_-C10 at pH 8, 19°C. PEG-6000 is not present.

1. Patchornik, G., et al., *Cryo-TEM structural analysis of conjugated nonionic engineered-micelles.* Soft Matter, 2014. **10**(27): p. 4922-8.
